# Umbilical pilonidal sinus: a rare cause of umbilical discharge

**DOI:** 10.1111/ans.17691

**Published:** 2022-04-06

**Authors:** Bushra Othman, Vinna An

**Affiliations:** ^1^ Epworth Eastern Private Hospital Epworth HealthCare Melbourne Victoria Australia; ^2^ Box Hill Hospital Eastern Health Melbourne Victoria Australia; ^3^ Department of Medicine Nursing and Health Sciences Monash University Melbourne Victoria Australia; ^4^ Department of General Surgery Barwon Health Geelong Victoria Australia

General surgeons are all too familiar with sacrococcygeal pilonidal disease and its various manifestations. The term pilo‐nidal was coined in 1880 by R.M. Hodges, defined by pilus (hair) and nidus (nest).^1^ He described certain elements that are necessary to support this diagnosis; a congenital coccygeal dimple, abundant pilous development (associated with adult age) and predominantly in the male sex. Case reports on umbilical pilonidal sinus (UPS) emerged in the early 19th century, and in 1956 a case of a young hirsute man with an infected umbilicus containing hairs was described by Patey and Williams and published in The Lancet.^2^ They studied the orientation of hairs within the sinus and concluded that the friction between the clothing and the abdominal wall drive loose hairs, root first, into the orifice of the sinus. This strengthened the theory that pilonidal sinus is an acquired disease. Other reported sites of pilonidal sinus affliction include the nose, axilla, breast and inter‐digital spaces of barbers and hairdressers.

A 62 year old man presented to his GP with complaints of pain around his umbilicus and purulent discharge. Over the last few months, he had trialled multiple courses of oral antibiotics, which would settle the infection for a short period, but then the symptoms would recur. There was no previous surgical history. An abdominal ultrasound was performed, and this showed an isoechoic ovoid focus tracking towards the peritoneum, measuring 8 × 9 × 7 mm (Fig. 1). There was some associated vascularity within this focus, but no obvious hernia.

**Fig. 1:**
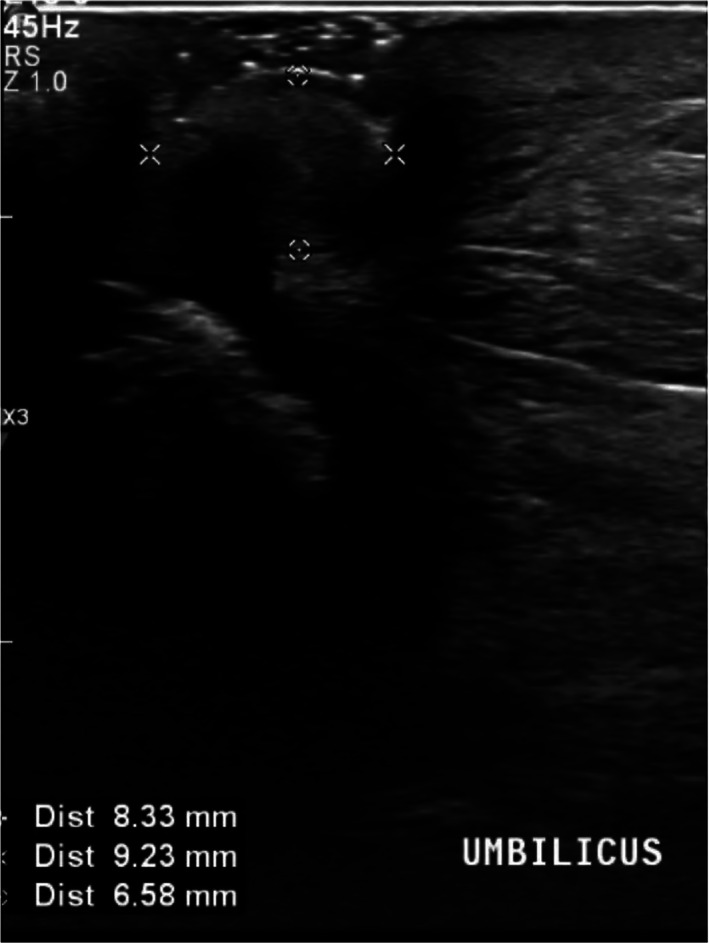
Ultrasound image of the umbilicus showing an isoechoic ovoid focus measuring 8 × 9 × 7 mm, with no associated hernia.

The patient proceeded to surgery for excision of the cyst. Examination of the abdomen showed a deep umbilicus with purulent discharge and surrounding hair, and minor skin erythema secondary to dressings due to the constant discharge (Fig. 2(a)). During intraoperative dissection, an abscess cavity containing hair and solidified sebaceous material was found (Fig. 2(b)). The sinus tract and abscess cavity was completely excised down to the level of the fascia. Entry into the peritoneal cavity showed no obvious extension towards the bladder or any other intraabdominal organ. The fascia and overlying skin were primarily closed. Histopathology confirmed skin with extensive ulceration and granulation tissue, and an organizing abscess with firm sebaceous material and hair.

**Fig. 2:**
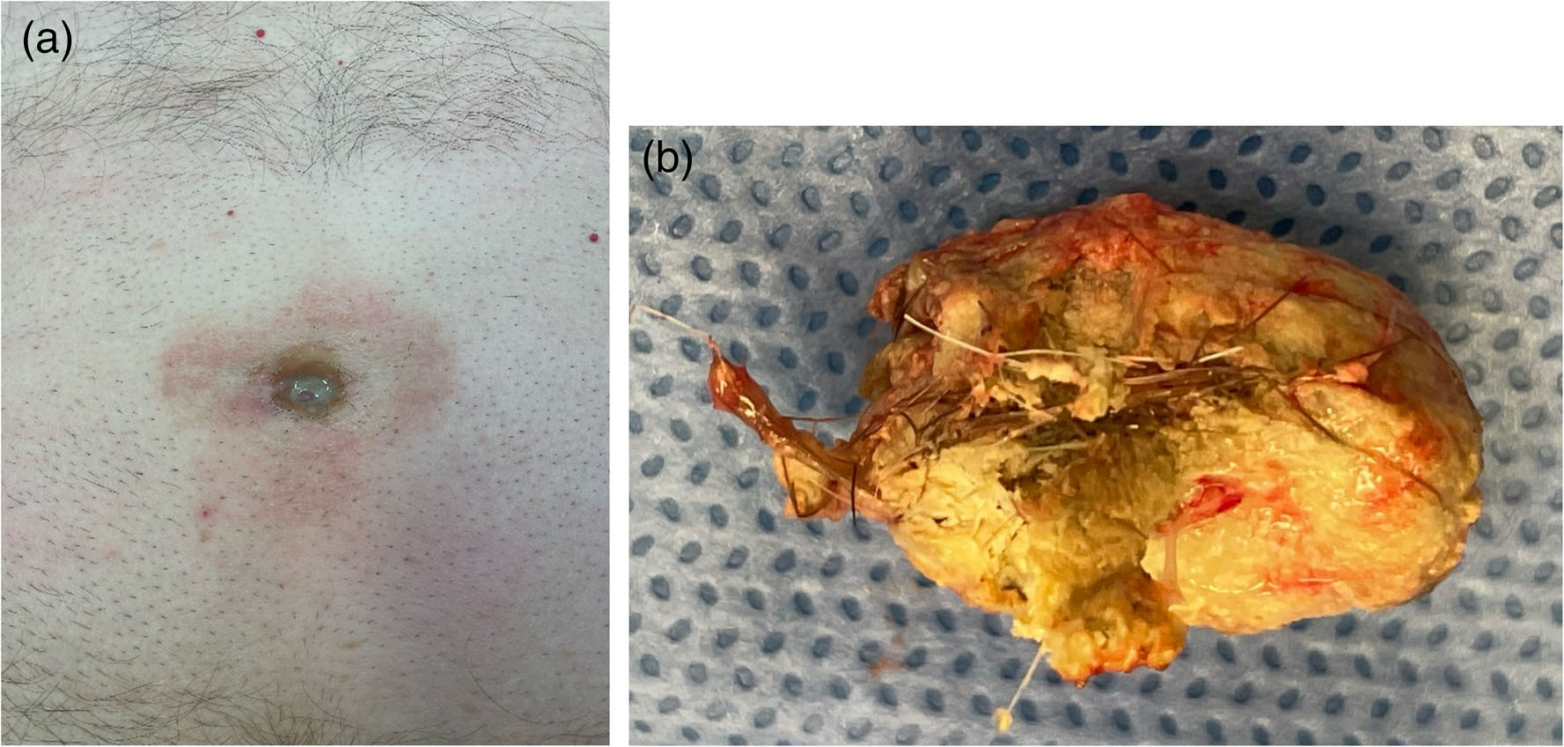
Intraoperative images demonstrating (a) deep navel with purulent discharge, skin erythema from dressings, and surrounding hair (shaved around the umbilicus for the procedure), and (b) contents of the abscess cavity (sebaceous material and hair).

Umbilical discharge is uncommon in the adult population, and especially in patients with no previous surgical history. Crucial differential diagnoses include umbilical pilonidal sinus, urachal remnant or omphalomesenteric duct remnant. The main predisposing factors for UPS include young age (twenties and thirties), male gender, hirsutism with the distribution of the distal ends of hairs pointing towards the umbilicus, a deep navel, and to a lesser degree obesity, hyperhidrosis and poor personal hygiene.^3^, ^4^ The umbilicus is on the belt line where clothes fit tightly and twisting of the trunk occurs, thereby increasing the risk of friction between hairs and the umbilicus predisposing hairs to getting caught. This triggers an inflammatory response resulting in oedema that further narrows the umbilical orifice, hence forming a sinus. The most common presenting complaint is a chronic discharging sinus at the umbilicus.^5^

Treatment options vary for patients suffering with UPS. Some studies have reported on successful conservative management of the disease with removal of hairs from the sinus as well as the surrounding hair, antibiotics, washing of the sinus with iodine and drainage of any concurrent abscess under local anaesthetic.^6^ Others advocate for a surgical approach, either as excision of the sinus and hair tufts, or complete excision of the umbilicus. Wounds can either be primarily closed or left to heal with secondary intention. Ponten and colleagues describe a treatment protocol for patients suffering from symptoms possibly caused by UPS (Fig. 3).^7^

**Fig. 3:**
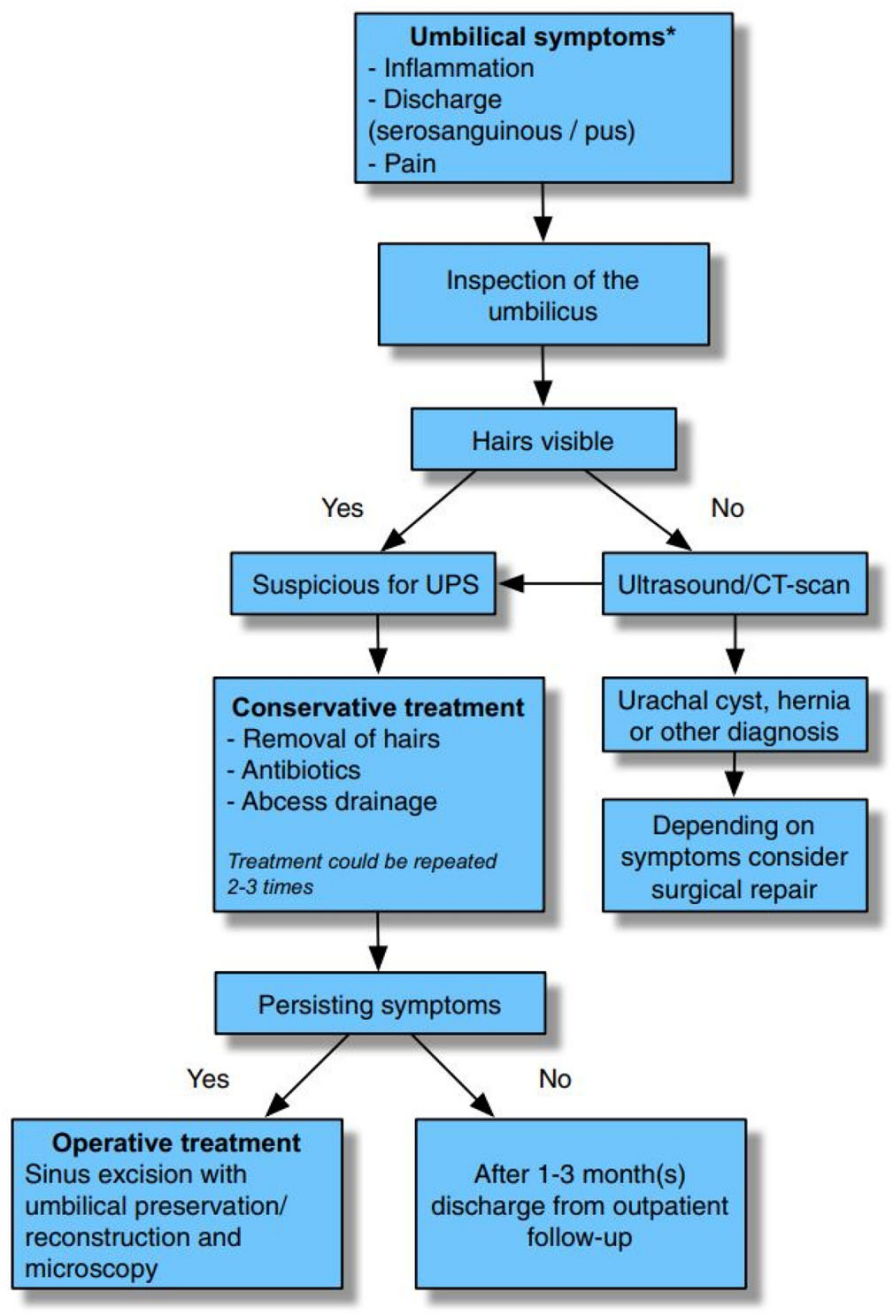
A treatment protocol for patients who suffer from symptoms possibly related to UPS.

The diagnosis of UPS is frequently overlooked in routine clinical practice because of lack of awareness. Persistent umbilical discharge that does not resolve with medical management should proceed to definitive management with surgical excision.

Informed consent was obtained from the patient prior to publication.

## Author contributions

**Bushra Othman:** Conceptualization; data curation; writing – original draft; writing – review and editing. **Vinna An:** Supervision; writing – review and editing.
